# Beyond patient contact: combined short- and long read sequencing reveals continuous occurrence of genomically related carbapenemase-producing Enterobacterales and plasmid mobility in a hospital, Germany, 2018 to 2021

**DOI:** 10.2807/1560-7917.ES.2025.30.23.2400590

**Published:** 2025-06-12

**Authors:** Nora Helke Leder, Monika Cristofolini, Sandra Ehrenberg, Mara Lohde, Christian Brandt, Mathias W Pletz, Frank Kipp, Claudia Stein

**Affiliations:** 1 Institute for Infectious Diseases and Infection Control, Jena University Hospital, Jena, Germany; 2 Hygiene and Infection Control, BG Hospital Bergmannstrost Halle, Halle, Germany; 3 Medizinische Universität Lausitz – Carl-Thiem-Klinikum Cottbus, Cottbus, Germany; 4 InfectoGnostics Research Campus, Centre for Applied Research, Jena, Germany

**Keywords:** plasmid analysis, core-genome, surveillance, infection control, transmission, hospital-acquired

## Abstract

**BACKGROUND:**

Carbapenemase-producing Enterobacterales (CPE) frequently cause nosocomial outbreaks. To investigate these, tracing focused on patients with related CPE strains and spatiotemporal contact (e.g. contact with each other in a room or on a ward during overlapping periods) has limitations. Moreover, as widely available molecular typing methods cannot detect plasmid-related transmissions, carbapenemase gene transfer across enteric bacteria through plasmids in hospitals remains poorly understood.

**AIM:**

Because whole-genome sequencing (WGS), particularly long-read sequencing, can offer insights into bacterial relationships both at core-genome and plasmid levels, we tested its utility, using VIM-CPE as example, to investigate plasmid and CPE spread in a hospital beyond outbreaks.

**METHODS:**

We included inpatient episodes from 2018 to 2021 involving *bla*_VIM-_bearing CPE isolates. Short- and long-read WGS data were combined with patient movement information to identify genomically related hospital-acquired VIM-CPE and putative transmission routes.

**RESULTS:**

Among 43 included inpatient episodes, 27 isolates were hospital-acquired, with 23 genomically related based on core-genome or plasmid analyses. For 14 of these 23 isolates, patient movement data supported suspected transmission events. Plasmid and core-genome level analyses revealed that most transmission events did not temporally concur, occurring over up to 33 months. Thus, conventional infection tracing methods focusing on concurrent spatiotemporal contact missed a substantial proportion of transmission events.

**CONCLUSION:**

With our findings, we advocate for broader epidemiological investigations of temporal connections if genomic data suggest relatedness. We emphasise considering plasmid transfer alongside analyses of core-genome relatedness of bacteria beyond patient contact events to study CPE and resistance spread, and guide infection control strategies.

**Key public health message**

**What did you want to address in this study and why?**

Carbapenemase-producing Enterobacterales (CPE) are bacteria with resistance to last-resort antibiotics that can cause severe infections. They can be transmitted between hospitalised patients. Moreover, genes responsible for antibiotic resistance of CPE can disseminate among bacteria through plasmids. Here, we combined short- and long read whole genome sequencing (WGS), to investigate CPE transmission routes and plasmid transfers in a hospital.

**What have we learnt from this study?**

Investigations guiding strategies to control CPE transmissions in hospitals, usually focus on outbreaks among patients directly in contact with each other, or with a common space during overlapping periods. With WGS analysis beyond outbreaks, we repeatedly uncovered related CPE strains over 4 years in patients without contact, who occupied common rooms or wards in separate times. Of 23 related CPE, six likely acquired resistance genes through plasmids.

**What are the implications of your findings for public health?**

Without WGS, some connection between patients and transmission routes would have been missed. To better detect CPE dissemination in hospitals, WGS should be used to trace both bacterial strains and resistance genes. This would allow more comprehensive monitoring of the spread of CPE and resistance in hospitals, including through or among patients without concurrent contacts or exposures, supporting more effective control measure development.

## Introduction

Management of carbapenemase-producing Enterobacterales (CPE) poses serious challenges for healthcare professionals and remains a top priority in the efforts to curb the spread of antimicrobial resistance [1]. Recent years have seen a concerning increase in CPE associated with healthcare [2]. Up to 16.5% of CPE-colonised patients progress to clinical infection [3], associated with heightened mortality [4], a substantial burden of disease [5], and increased healthcare costs [6]. Contact with healthcare settings significantly amplifies the risk of CPE acquisitions [7], since CPE can persist on surfaces or within water systems of hospitals for up to 5 years [8], facilitating transmission through reservoirs in the patient environment over prolonged periods [9]. Such persistence, as well as lapses in hygiene practices can lead to healthcare-associated outbreaks [10]. Outbreaks are defined by the World Health Organization as the occurrence of more cases of disease than normally expected [11]. National definitions, such as those in Germany, align with this concept while emphasising the necessity of detecting infections to declare an outbreak [12]. However, as not all patients colonised with CPE develop infections [3], asymptomatic carriers can contribute to the spread of CPE before an outbreak occurs [13].

Integrating whole genome sequencing (WGS) into infection prevention practices leverages the high resolution of this method to uncover genomic links not only between infected patients, but also between asymptomatic carriers [13,14].

To evaluate transmission routes established by WGS, most studies focus on concurrent spatiotemporal contact between patients [10,15,16] while only a few consider temporally non-concurrent exposure [17,18]. Consequently, the extent of such transmissions and their relevance for routine infection prevention are insufficiently explored.

A well-established tool for assessing nosocomial transmission likelihood is WGS-based core-genome analysis [17,19]. This approach allows to explore relatedness of CPE by vertical transmission, thereby supporting investigations of parent and offspring strains’ spread. Nevertheless, it is also important to acknowledge that antibiotic resistance genes, such as carbapenemase genes in Enterobacterales, can not only disseminate through vertical transfer (i.e. the genes spread as the bacteria that bear them propagate) but also through horizontal gene transfer (i.e. the genes spread among bacteria) via mobile genetic elements. These elements, including plasmids, can even cross species barriers [20]. Thus, plasmids can contribute to the rapid dissemination of resistance mechanisms across bacterial populations [21]. Tracing mobile genetic elements, however, has not been included in routine surveillance. Consequently, the extent of plasmid-related transfer of resistance mechanisms remains uncertain.

Our study investigates the advantage of continuously monitoring, beyond outbreaks, the genetic relatedness of CPE and their mobile genetic elements underlying resistance. Through a longitudinal analysis of nosocomial clusters of Verona integron-encoded metallo-β-lactamase (VIM)-CPE, we also assess the utility of WGS core-genome and plasmid-level data combined with epidemiological and patient movement information, to further uncover potential transmission routes within a hospital.

## Methods

### Study design

We conducted a longitudinal study at our 1,400-bed tertiary care hospital during a period ranging from 1 January 2018 to 31 December 2021. Isolates were obtained from clinical specimens taken for diagnostics and from screening samples.

Screening was performed according to German guidelines [22] within the first 2 days of admission via rectal swab or at the site of chronic wounds or previous CPE detection if patients had travelled abroad or stayed at a healthcare facility in Germany within the last 12 months. If CPE were detected, measures to prevent transmissions included hand hygiene, personal protective equipment, environmental cleaning and disinfection, and contact precautions like isolation and individual care. Contact patients admitted to the same room as the index patient for at least 24 hours were screened once via rectal swabs to detect transmissions. When patients were readmitted, each admission was treated as a new inpatient episode, and CPE isolates were reassessed accordingly.

Patient movement data for each inpatient episode as well as data on age, sex (collected as a binary male/ female variable), medical procedures and type of ward were retrieved from the medical records.

In the current investigation, we included all hospitalisation episodes during the study period, of patients testing positive for VIM-CPE at any point during their admission. VIM-CPE detected within the first 2 days after admission were designated as community-acquired, VIM-CPE isolates first detected after the second day of admission were classified as hospital-acquired.

### Microbiology and genomic analysis

#### Microbiology

The samples were streaked onto Columbia sheep blood agar, Drigalski lactose agar (Oxoid, Thermo Fisher Scientific, Wesel, Germany), and CHROMagar ESBL/KPC (Mast Diagnostica, Reinfeld, Germany) for CPE screening. If morphologically different colonies were observed on the culture plate, these colonies were individually isolated and further analysed. Species identification was performed by Vitek MS (Shimadzu, bioMérieux, Nürtingen, Germany) using the Vitek IVD database. Isolates were tested for carbapenemase family genes by the eazyplexSuperBug CRE assay (AmplexDiagnostics, Gars-Bahnhof, Germany) according to the manufacturer’s protocol. When CPEs were detected multiple times in the same patient, and isolates originated from the same sample type, represented the same species and exhibited identical resistance profiles in the phenotypic or genotypic analysis, only the first isolate was subjected to genomic analysis.

#### Short-read sequencing

High-throughput WGS and subsequent data analysis were then performed with MiSeq Illumina as described before [14]. Genomic DNA was paired-end sequenced with a MiSeq Reagent kit v2 2×150 bp (Illumina San Diego, United States (US)) with an average insertion size of 300 bp.

The resulting reads were quality-trimmed and *de novo–assembled* using the Velvet algorithm integrated into the Ridom Seqsphere + software version 7 (Ridom GmbH, Muenster, Germany). For *Klebsiella pneumoniae,* we used a public core-genome multilocus sequence typing (cgMLST) (reference genome: *K. pneumoniae* subsp. *pneumoniae* NTUH-K2044 DNA, complete genome). For *Citrobacter freundii, Enterobacter cloacae*, and *K. oxytoca* an ad hoc cgMLST was established according to Ridom SeqSphere + guidelines. For this purpose, *C. freundii* strain 18–1 (GenBank: NZ_CP022273.1), *E. cloacae* subsp. *cloacae* ATCC 13047 (GenBank: NC_014121.1) and *K. oxytoca* strain CAV1374 (GenBank: NZ_CP011636.1) were selected as seed genomes. Minimum-spanning tree analyses were performed based on the determined allelic profiles using the Ridom Seqsphere + software with the parameter ‘pairwise ignore missing values’. We defined a cluster of clonal isolates if the cgMLST revealed a difference of ≤ 10 alleles for *C. freundii* and *E. cloacae* and ≤ 15 alleles for *K. oxytoca* and *K. pneumoniae* [23].

To further characterise the genomic distance between the isolates, we also performed a core-genome single nt polymorphisms (cgSNPs)-analysis. Within the Ridom Seqsphere + software version 7 (Ridom GmbH, Muenster, Germany), we used the ‘Find SNVs in Distance Columns’-Tool with the same reference genomes as in the cgMLST-analysis. Single nt variation (SNV)-distance between the isolates was calculated based on all columns with the setting ‘pairwise ignore missing values’.

For the detection of antimicrobial resistance genes, the ResFinder tool (v4.1) was used [24]. The sequencing data are publicly available at the European Nucleotide Archive (accession numbers: PRJEB59013, PRJEB43549, PRJEB50554, PRJEB42370, PRJEB43550, PRJEB60429).

#### Long-read sequencing

To assess the impact of plasmids on resistance gene transmission, we performed additional sequencing using long-read WGS (Oxford Nanopore Technologies, Oxford, United Kingdom (UK)). We selected all isolates from the final year of the study period (2021) to examine the current plasmid landscape associated with VIM-producing CPE in our hospital. The selected isolates underwent long-read sequencing using the Oxford Nanopore Technology. Extraction of DNA was performed with the ZymoBIOMICS DNA Microprep kit (ZYMO Research, Irvine, US), and for library preparation the Ligation Sequencing Kit (SQK-LSK-110) (Oxford Nanopore Technologies, Oxford, UK) was used. For sequencing, the library was loaded on a FLO-MIN105 version R9.4 flow cell. Taxonomic classification was conducted using Genome Taxonomy Database (GTDB)-Tk (version 1.6.0) software toolkit and the GTDB database (version r202).

#### Plasmid analysis

Plasmids were identified based on the presence of *bla*_VIM_ and Inc groups and contig size. Because long-read Nanopore data were only available for isolates from 2021, we used the available Nanopore data from these isolates to identify plasmid-contigs within the Illumina-only data from isolates obtained in the previous years. Illumina short-reads were mapped on each long-read plasmid contig with minimap2 (v2.24) (https://github.com/lh3/minimap2) and visualised as coverage plots using SAMtools (v1.14) (https://github.com/samtools/samtools). A coverage threshold of over 95% for the entire long-read sequence was set as the cutoff for similarity. If the short-read data contained the sequence identified in the long-read data, the isolate was assigned to the same plasmid as the reference. In cases where isolates’ short-read sequences matched with multiple long-read reference sequences, they were compared using Basic Local Alignment Search Tool (BLAST) + 2.13.0 with a cutoff of 95% coverage and 99% sequence identity to determine the number of distinct plasmids. Subsequently, distinct plasmids were compared pairwise using BLAST + 2.13.0 for further analysis.

### Transmission events

**Figure 1:**
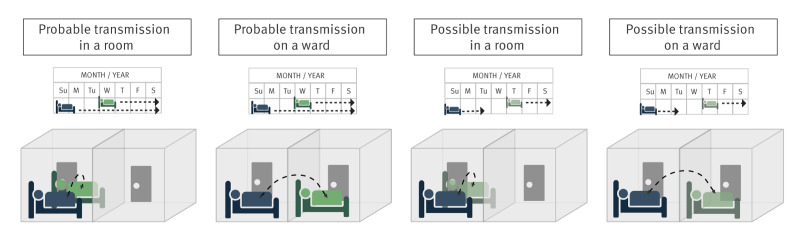
Characterisation of putative transmission events for hospital-acquired isolates with genomic connections, Germany, 2018–2021 (n = 27 hospital-acquired isolates)

Data on movement of patients with hospital-acquired VIM-CPE were compared with any inpatient episodes (community- or hospital-acquired) where isolates related on core-genome or plasmid level were detected. The plausibility of potential transmission events was verified based on the evaluation of microbiological findings from diagnostics and screening. To evaluate the likelihood of putative transmission events, four categories were established based on the probability of healthcare-related acquisition [25]. For this, we adapted the method of Voor In‘t Holt et al. [25], by applying the same dimensions (room, department/ward, and time) but modifying the scale of transmission likelihood categories as follows. The categories included from most to least likely: (i) ‘probable transmission in a room’, where patients shared the same room concurrently; (ii) ‘probable transmission in a ward’ where patients were admitted to the same ward at the same time; (iii) ‘possible transmission in a room’ where patients were admitted to a room previously occupied by a potential source patient, and (iv) ‘possible transmission in a ward’ where patients were admitted to the same ward without overlap in their stays (Figure 1). Patients not fitting the criteria for these groups were classified as having no identified transmission route.

F: Friday; M: Monday; T: Thursday; Tu: Tuesday; W: Wednesday; S: Saturday; Su: Sunday.

When VIM-CPE isolates from patients had a core-genome or plasmid level connection, the patients’ movements were analysed. Putative transmissions were categorised into four groups from most to least likely: ‘probable transmission in a room’ (transmission between patients concurrently sharing the same room), ‘probable transmission on a ward’ (transmission between patients concurrently admitted to the same ward), ‘possible transmission in a room’ (transmission between patients occupying the same room at distinct times), and ‘possible transmission in a ward’ (transmission between patients admitted to/in the same ward at non-overlapping times).

To distinguish between the transfer of resistant strains and plasmid-related transfer of carbapenemases, we applied the definitions by Marimuthu et al. [18]: Transfer of a resistant strain was deduced if the isolates involved were core-genome-related. On the other hand, plasmid-related transfer of the carbapenemase gene was inferred if the isolates had no observed core-genome relatedness but had the same plasmid sequence detected. The most probable transmission route was assigned to each isolate.

## Results

**Figure 2:**
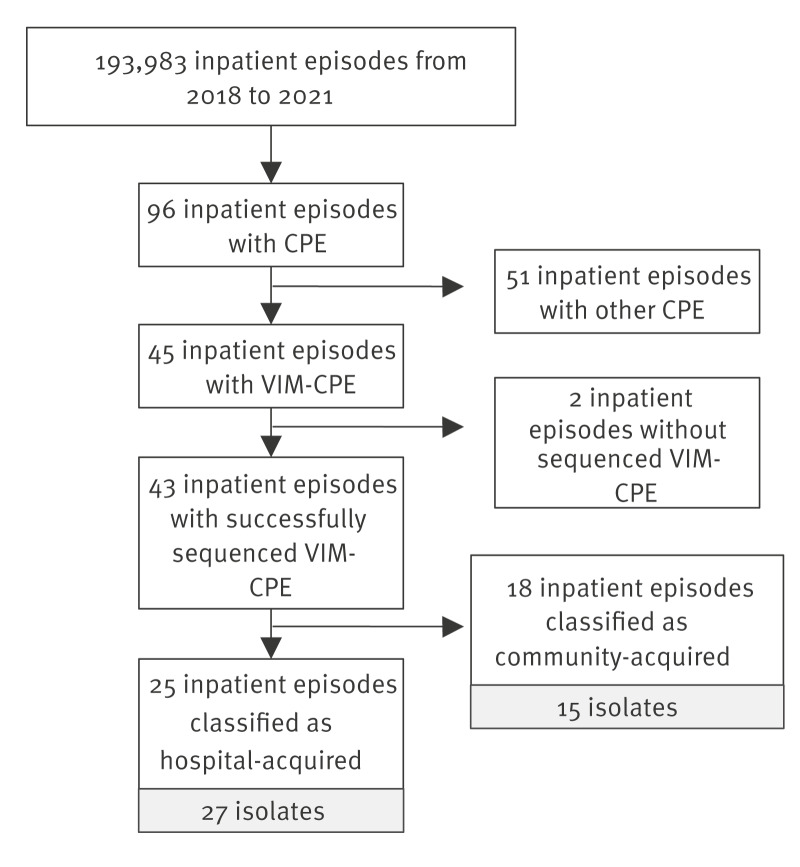
Flow diagram of VIM-CPE inpatient episodes included in the study, Germany, 2018–2021 (n = 43 selected)

CPE: carbapenemase-producing Enterobacterales; VIM: Verona integron-encoded metallo-β-lactamase.

During the study period, *bla*_VIM_ was the most prevalent carbapenemase among CPE within our hospital. We detected 45 inpatient episodes involving VIM-CPE and two were excluded due to the lack of sequencing result. A total of 43 inpatient episodes met the inclusion criteria (Figure 2). Eighteen inpatient episodes were classified as community-acquired. Notably, all but one patient had at least one previous stay at our hospital, but no further analysis of those previous admissions was performed. Twenty-five of the 43 inpatient episodes were categorised as hospital acquired. Two of the 25 patients involved in these inpatient episodes had two isolates each (including one patient with two different VIM-CPE species), resulting in 27 hospital-acquired isolates (Figure 2).

Flow diagram of inpatient episodes included, as well as inpatient episodes excluded due to the absence of VIM-carbapenemase and sequencing data, as well as classification as hospital- and community-acquired inpatient episodes and isolates, based on the VIM-CPE screening and diagnostic results within 2 days of admission.

The 25 patients with hospital-acquired VIM-CPE had a mean age of 73 years (range: 27–80) and consisted mainly of men (18/25). More than half were patients who had received surgery (17/25). The most common primary departments were vascular surgery, thoracic surgery, and internal medicine. Notably, most of them (18/25) received care in an intensive care unit (ICU), and three underwent organ or stem cell transplantation during admission.

### cgMLST analysis reveals transmission events with extended temporal gaps

All hospital-acquired isolates carried *bla*_VIM-1_. Furthermore, five isolates carried additional carbapenemases, including *bla*_OXA-1,_ *bla*_OXA-10,_ *bla*_OXA-162_, respectively. We detected hospital-acquired isolates in nine different *bla*_VIM_-bearing species (Table). Of these, the most frequently detected were *C. freundii*, *K. oxytoca*, *K. pneumoniae* and *E. cloacae*.

**Table:** Included isolates of VIM-CPE and distribution of hospital-acquired isolates among the different species, Germany, 2018–2021 (n = 42 isolates)

Species, n = 11	Number of VIM-CPE isolates, n = 42	Number of hospital-acquired VIM-CPE isolates, n = 27
*Citrobacter freundii*	19	12
*Klebsiella oxytoca*	7	5
*Klebsiella pneumoniae*	4	4
*Enterobacter cloacae*	4	1
*Enterobacter hormaechei*	2	1
*Citrobacter brakii*	1	1
*Citrobacter farmeri*	1	0
*Escherichia coli*	1	0
*Hafnia alvei*	1	1
*Klebsiella aerogenes*	1	1
*Providencia rettgeri*	1	1

CPE: carbapenemase-producing Enterobacterales; VIM: Verona integron-encoded metallo-β-lactamase.

**Figure 3:**
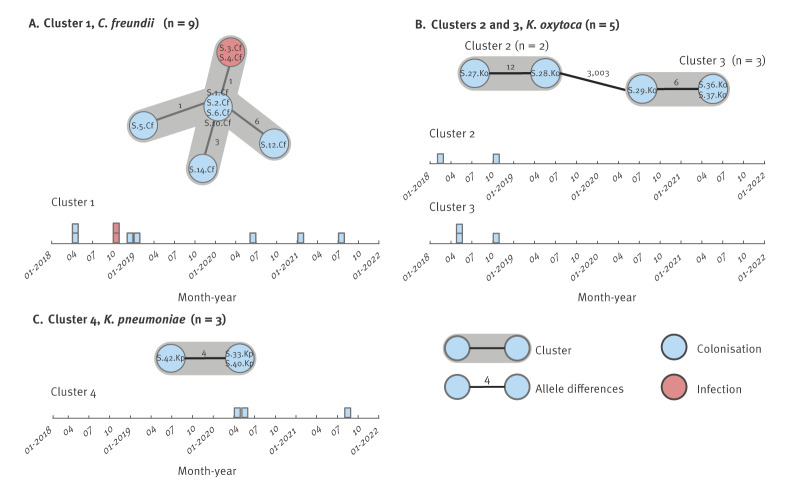
Genomic relationships of hospital-acquired VIM-CPE based on allelic differences in the cgMLST analysis, Germany, 2018–2021 (n = 17 hospital-acquired isolates)

Among the 27 hospital-acquired isolates, 17 were grouped into four clusters (clusters 1−4) within their respective species based on allele differences below the species-specific cluster thresholds defined in the methods section (Figure 3). The cgSNP-analysis confirmed the clusters found in the cgMLST-analysis with 0–12 SNPs between the isolates in the clusters. Details on the genomic relationships found based on SNP-differences are presented in Supplementary Material 1, Figure S1. Isolates in cluster 1 (*C. freundii*) were detected throughout the entire study period, while those in clusters 2, 3 (*K. oxytoca*), and 4 (*K. pneumoniae*) were detected throughout 7, 5 and 15 months, respectively (Figure 3).

Cf: *Citrobacter freundii*; cgMLST*:* core genome multilocus sequence typing; CPE*:* carbapenemase-producing Enterobacterales; Kp: *Klebsiella pneumoniae*; Ko*:* *K. oxytoca*; VIM: Verona integron-encoded metallo-β-lactamase.

For (A) *C. freundii*, (B) *K. oxytoca* and (C) *K. pneumoniae* isolates, the cgMLST-based relationships as well as epidemic curve are shown. Isolate names follow the pattern ‘S.consecutive number.species’. Each isolate is displayed in a circle. Isolates within the same circle have no allelic differences. Numbers on the lines represent the number of allelic differences. Clusters of clonal isolates are indicated by a grey background colour. In the epidemic curve, each isolate is represented by a rectangle, positioned along the X axis according to the month of VIM-CPE detection. The colours of the circles (in the clusters) and of the rectangles (in the epidemic curves) represent colonisation with CPE (blue) or clinical infection (red). Isolates S.36.Ko and S.37.Ko were detected in a single patient (patient 1).

An epidemiological analysis based on core-genome relatedness was conducted to investigate transmission routes and more detailed information on this analysis is available in Figures S2–S5 in Supplementary Material 1. Among the 17 hospital-acquired isolates within the four clusters, nine isolates from nine respective patients (S.3.Cf, S.4.Cf, S.28.Ko, S.5.Cf, S.10.Cf, S.33.Kp, S.6.Cf, S.12.Cf, and S.14.Cf), were found to have an epidemiological connection. Because multiple transmission events were revealed for each isolate, the acquisition of the nine hospital-acquired isolates could be explained by 15 potential transmission events.

Considering the most likely acquisition routes, we identified three probable transmissions on a ward, three possible transmissions in a room and three possible transmissions in a ward. Among the probable transmissions on a ward, one concerned S.38.Ko (community-acquired, data not shown) and S.28.Ko, and two could explain S.3.Cf and S.4.Cf (including one transmission from S.3.Cf to S.4.Cf and one from S.4.Cf to S.3.Cf, with each direction considered equally likely). Of the three possible transmissions in a room, one involved isolates S.3.Cf and S.5.Cf, one S.3.Cf and S.10.Cf, and one S.40.Kp and S.33.Kp). Regarding the three possible transmissions on a ward, one involved isolates S.5.Cf and S.6.Cf, one S.2.Cf and S.12.Cf, and one S.11.Cf (community-acquired, data not shown) and S.14.Cf, as illustrated in Supplementary Material 1 Figure S8.

The analysis revealed substantial temporal gaps between patients harbouring isolates belonging to the same cgMLST cluster who were involved in suspected transmission events. Examination of these events showed a median time gap within possible transmission events of 7 months, ranging from 1 to 33 months. Notably, only eight of all 25 patients with hospital-acquired VIM-CPE were admitted to the hospital at the same time as a patient harbouring a core-genome-related isolate, otherwise there was no overlap in admission to the hospital within the clusters.

### Putative plasmid transfer across multiple species uncovers hidden transmission events

**Figure 4:**
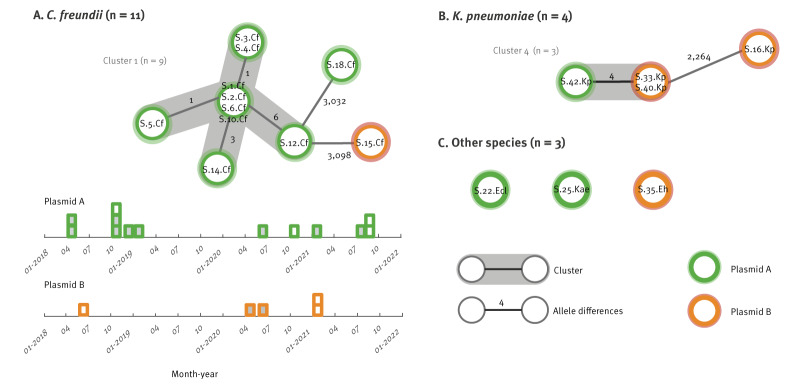
Plasmid distribution for hospital-acquired VIM-CPE based on short and long-read sequencing, Germany, 2018–2021 (n = 18 hospital-acquired isolates)

By combining short- and long-read sequencing, we identified two distinct plasmids in multiple hospital-acquired isolates. Plasmid A was present in 13 hospital-acquired isolates across four species (Figure 4). It is an IncHI1B plasmid with a length of 384 kbp and encodes the VIM-1 β-lactamase, as well as trimethoprim-sulfamethoxazole (sul1), quinolone (qnr-S1), and aminoglycoside (aadA1p.m.) resistance genes. Plasmid B, with a length of 157 kbp, was found in five hospital acquired isolates across three species (Figure 4). It belongs to the IncA group and harbours various β-lactamases such as sulfhydryl variable (SHV)-12, oxacillinase (OXA)-10, and VIM-1, along with resistance genes for trimethoprim-sulfamethoxazole, tetracycline, chloramphenicol, and aminoglycosides.

Cf: *Citrobacter freundii*; cgMLST: core genome multilocus sequence type; CPE: carbapenemase-producing Enterobacterales; Ecl: *Enterobacter cloacae*; Eh: *E. hormaechei*; Kae: *Klebsiella* *aerogenes*; Kp: *K. pneumoniae*; VIM: Verona integron-encoded metallo-β-lactamase.

cgMLST-based genomic relations as seen in Figure 3 for (A) *Citrobacter freundii*, (B) *Klebsiella pneumoniae*, and (C) other species. The coloured glow around the circles indicates the presence of plasmid A (green) or B (orange) that are present in more than one isolate. Plasmids other than A and B, present in only one isolate, are not shown. In the epidemic curve, each isolate is represented by a rectangle indicating the month of VIM-CPE detection. Gray rectangles indicate a core-genome relationship. S.15.Cf and S.16.Kp were detected in a single patient (patient 2).

We examined epidemiological connections among isolates harbouring plasmids A and B. Among the 27 hospital-acquired isolates, all nine core-genome-related isolates of *C. freundii* cluster 1 exhibited relatedness at the plasmid level (Plasmid A) as well. Within cluster 4, plasmid A and B were present among the *K. pneumoniae* isolates (Figure 4).

Genomic relatedness only at the plasmid level was observed in six additional hospital-acquired isolates (Figure 4). Between the patients with isolates harbouring plasmids A or B, five plasmid transfers could have been facilitated by 12 potential transmission routes. The five transfers respectively involved two isolates with plasmid A (S.22.Ecl and S.18.Cf) and three isolates with plasmid B (S.40.Kp, S.15.Cf, S.16.Kp). Detailed information can be obtained from Supplementary Material 1 Figures S6 (for plasmid A) and S7 (for plasmid B). Notably, the patients respectively carrying isolates S.33.Kp and S.40.Kp, as well as the patient from whom S.15.Cf and S.16.Kp were isolated stayed in the same room over a period of 8 months, shortly before the detection of plasmid B. The median time gap within plasmid-related epidemiologic transmission events was 23 months, ranging from 1 to 31 months. Of the six isolates with a genomic connection exclusively on the plasmid level, only three patients were admitted to the hospital at the same time as a patient harbouring a plasmid-related isolate, otherwise there was no overlap in admission to the hospital.

## Discussion

In addition to the well-known intra-hospital transmission of VIM-CPE-strains by concurrent spatiotemporal contact between patients, our research suggests endemic persistence of CPE within our hospital and multispecies plasmid-related transfer of resistance genes. These findings emphasise the role of WGS in identifying previously unrecognised transmission routes for CPE in hospitals, particularly those through temporally non-concurrent exposure.

We implemented a WGS-based surveillance approach for CPE following a VIM-CPE outbreak in our hospital in 2016 [26]. Our findings align with previous studies that point out the high-resolution capabilities of WGS in including or excluding inpatient episodes from infection control investigations in hospitals based on genomic relatedness [15,27]. The clarity of molecular findings can be used to establish connections between sporadic occurrences and uncover previously undetected transmission routes.

Due to the lack of a rise in CPE-infections in our hospital and prolonged gaps between inpatient episodes, no outbreak definition was met. Nevertheless, we reported a high core-genome relatedness among hospital-acquired CPE. We could demonstrate the congruence between the cgMLST and cgSNP analysis, which has been suggested previously for other Enterobacterales species [23,28]. The SNP difference within our clusters aligns with reported SNP differences between epidemiologically linked Enterobacterales isolates [27,29], though comparison is hard due to the influence of the chosen reference genome on the resulting number of SNPs [30].

Interestingly, our systematic analysis of inpatient episodes with genomically related isolates identified only three probable transmissions of VIM-CPE through concurrent spatiotemporal contact between patients. Our data further suggest that possible transmission through temporally non-concurrent ward or room exposure is the most common route of transmission, accounting for six of nine transmissions for core-genome-related isolates. Studies in hospitals on CPE transmissions without concurrent spatiotemporal contact support our findings [17,18], one even identifies these as significant risk factors for CPE acquisition [18]. In our study, infection control investigations focusing solely on concurrent spatiotemporal contact between patients would have missed most transmission events. While the proportion of missed events may vary across settings, extending the temporal scope of routine infection control investigations to several months, if WGS suggests relatedness between isolates, could significantly improve the detection of transmission routes in similar contexts. Further studies are needed to determine the optimal time span for investigation. This time frame must be practical and feasible for routine infection prevention efforts.

Another aspect that is often overlooked in conventional infection control investigations is the role of multispecies plasmid-related transfer of resistance genes. Accurate reconstruction of plasmids requires long-read sequencing [31], which we incorporated into our study by combining short- and long-read sequencing methods. This mapping strategy, which uses plasmid sequences identified from long-read data as references to detect their presence in short-read sequences, is particularly useful in settings where long-read sequencing is not available for all isolates, as demonstrated in this study. Similar approaches were previously used in other clinical settings but with varying sequence coverage cut-offs [10,32]. 

The use of WGS for plasmid-mediated multispecies outbreaks in hospitals has been previously described [21], but the role of plasmids in spreading antimicrobial resistance genes outside of outbreak scenarios remains underexplored. In our study, we identified only a few highly similar plasmid sequences from bacteria carried by patients over extended periods, underscoring the persistence and potential clinical impact of these elements. Notably, plasmids within the same Inc-families (IncHI1B and IncA) were previously identified in multispecies outbreaks [32,33], indicating their clinical relevance in CPE transmission. While VIM-1-carrying plasmids have been sporadically reported in Germany [34], the available data are insufficient to assess their potential dissemination within the country.

As we could show before for extended-spectrum β-lactamase (ESBL)-producing Enterobacterales [35], heterogeneity among resistant strains was higher compared with the plasmids. This suggests that plasmids may play an important role in disseminating resistance genes across diverse bacterial populations. Compared with a recent study from Singapore [18], we observed a lower rate of plasmid-related transfer of resistance genes than transfer of resistant strains. However, further investigation is required to confirm this trend and to better understand the dynamics of plasmid-related gene transfer.

While the majority of connections on core-genome level were strengthened by the plasmid analysis, we uncovered additional transmission routes, particularly between five isolates, that were missed by infection prevention investigations without WGS and those relying solely on core-genome-based approaches. This suggests the need for comprehensive genomic surveillance strategies that include long-read sequencing to accurately detect and reconstruct plasmids. The complexity of data, stemming from both sequencing and patient movement information, presents a challenge when analysing transmissions. The development of software-based solutions will be crucial for the future implementation of such complex analyses in routine workflows.

This study has some limitations. We relied on rectal swabs as our primary detection tool, although studies have shown that pre-analytic parameters can greatly impact the significance of these tests [36]. With our approach, we could not draw conclusions about the direction of the transmissions for patients with direct contact. While strict contact precautions were implemented following the detection of CPE in patients (e.g. isolation in single rooms), and contact patients (before confirmation of CPE status) were screened as part of our protocol, the possibility of undetected transmission pathways cannot be excluded. Nevertheless, undetected CPE carriage in patients may account for the absence of some links within transmission clusters. Additionally, CPE carriage among healthcare personnel represents a potential route of transmission. However, existing studies, such as the findings by Badinski et al. [37], indicate a low colonisation rate of CPE among healthcare workers. Furthermore, patient transfer between healthcare facilities [38] and lapses in adherence to infection control measures are identified as significant contributors to the spread of CPE [39]. For the detection of transmission routes within our hospital, we focused on hospital-acquired isolates. If community-acquired isolates showed genomic connections (data not shown), the inpatient episode was considered as source, but due to the frequent previous admissions, we neither can rule out nor rule in undetected acquisition during one of these previous admissions for isolates labelled as community-acquired. Considering we did not identify transmission events for all hospital-acquired isolates, further investigations could focus on transmissions outside the ward by comparing procedures. Since only a few patients were admitted to the hospital at the same time as genomically related cases, transmissions outside of wards cannot explain CPE acquisition for all isolates without a transmission route in our analysis. Additionally, the suspected transmission routes need validation. We did not include environmental samples in our study, but analysing such samples, especially from the rooms where transmission events were suspected could confirm suspected transmission routes.

## Conclusion

With this study from 2018 to 2021, we uncovered that among 43 inpatient episodes with VIM-CPE, 27 isolates were likely hospital-acquired. Among these 27, 23 isolates were genomically related based on core-genome or plasmid analyses, with patient movement data supporting suspected transmission events in 14 (including nine cgMLST- and five plasmid-related isolates). Based on these results, we advocate to broaden investigation of temporal connections between genetically related CPE isolates as part of surveillance programmes in hospitals. Our data underscore the significance of analysing plasmids alongside the degree of relatedness among bacterial strains in infection control efforts. This highlights the necessity of streamlining such analyses in routine diagnostics and incorporating the results into clinical practice. With these findings in mind, it may be worthwhile to use long-read WGS data analysis results to further guide strategies aiming to control cabapenemase-gene transfer in hospitals.

## Data Availability

Whole-genome sequencing (WGS) data are available at the European Nucleotide Archive (ENA) under the following study accession numbers: PRJEB59013, PRJEB43549, PRJEB50554, PRJEB42370, PRJEB43550, PRJEB60429.

## Supplementary Data

2764000 Supplementary Material 1
