# Supplementary material for: Beyond patient contact: combined short- and long read sequencing reveals continuous occurrence of genomically related carbapenemase-producing Enterobacterales and plasmid mobility in a hospital, Germany, 2018 to 2021
Source: Euro Surveill. 2025 Jun 12;30(23):2400590. doi: 10.2807/1560-7917.ES.2025.30.23.2400590 (PMC12164281; doi:10.2807/1560-7917.ES.2025.30.23.2400590)
Supplement: Supplementary Material 1 [file 2400590_SupplementaryMaterial_1.pdf]

Supplemental Material 1

This supplementary material is hosted by *Eurosurveillance* as supporting information alongside the article "Beyond patient contact: combined short- and long read sequencing reveals continuous occurrence of genomically related carbapenemase-producing Enterobacterales and plasmid mobility in a hospital, Germany, 2018 to 2021", on behalf of the authors, who remain responsible for the accuracy and appropriateness of the content. The same standards for ethics, copyright, attributions and permissions as for the article apply. Supplements are not edited by *Eurosurveillance* and the journal is not responsible for the maintenance of any links or email addresses provided therein.

Content

Supplementary Figure S1: SNP-Analysis 2

Supplementary Figure S2: Patient Movement Data and Transmission Events in Cluster 1 3

Supplementary Figure S3: Patient Movement Data and Transmission Events in Cluster 2 4

Supplementary Figure S4: Patient Movement Data in Cluster 3 5

Supplementary Figure S5: Patient Movement Data and Transmission Events in Cluster 4 6

Supplementary Figure S6: Patient Movement Data and Transmission Events for Plasmid A 7

Supplementary Figure S7: Patient Movement Data and Transmission Events for Plasmid B 8

Supplementary Figure S8: Characterisation of putative transmission events for hospital-acquired isolates with genomic connections 9

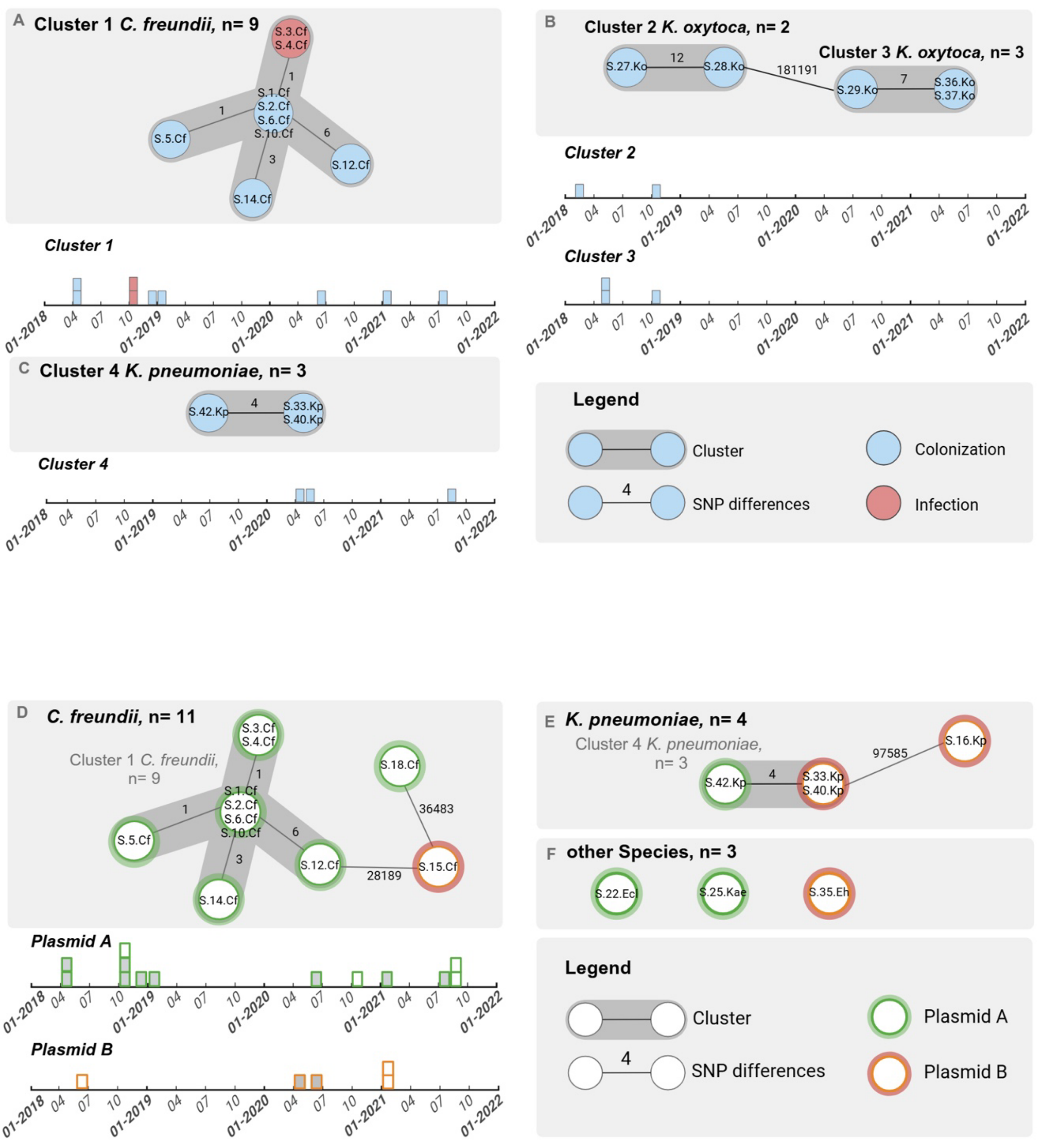

Supplementary Figure S1: Genomic relationships of hospital-acquired VIM-CPE based on SNP-differences in the SNP-analysis.

For *C. freundii, K. oxytoca, K. pneumoniae* and isolates from other species (A-F), SNP-based relationships as well as epidemic curve are shown. Isolate names follow the pattern of S.consecutive number.species. Each isolate is displayed in a circle. Isolates within the same circle had no allelic differences. Clusters of clonal isolates are indicated by a grey background color. The colors of the circles represent colonization with CPE (blue) or clinical infection (red). The colored glow around the circles indicates the presence of plasmid A (green) or B (orange) that are present in more than one isolate. Plasmids present in only one isolate are not shown. In the epidemic curve, each isolate is represented by a rectangle indicating the month of VIM-CPE detection.

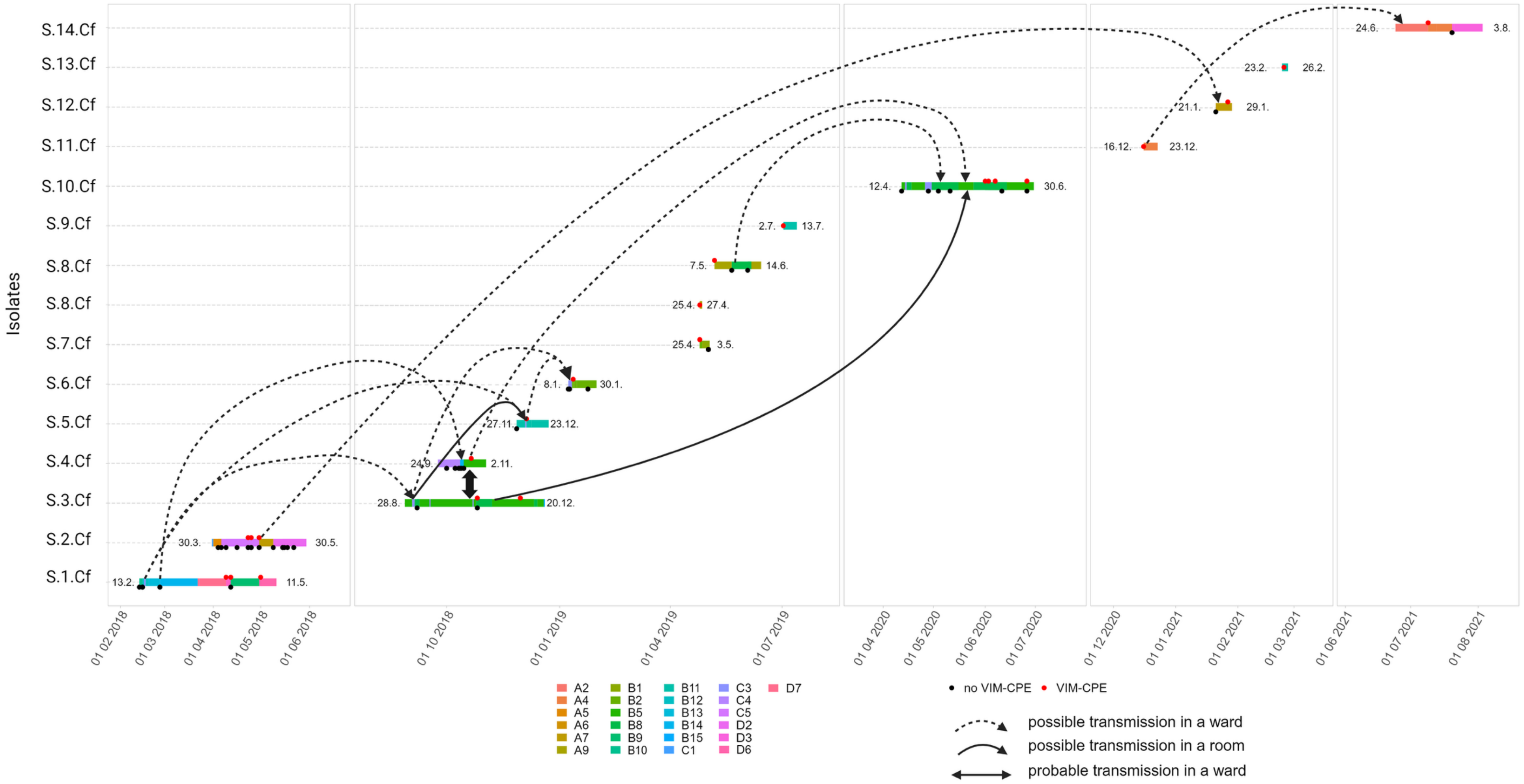

Supplementary Figure S2: Patient Movement Data and Transmission Events in Cluster 1

The figure illustrates patient movement data over time for each inpatient episode in cluster 1 (*C. freundii*), identified by sequencing numbers. Different colored bars represent the wards patients were admitted to, with the length of each bar indicating the length of stay. Dates adjacent to the bars denote admission and discharge. Wards were grouped into categories A, B, and C based on their proximity within the building, and then assigned random numbers. Wards labeled with a prefix of "D" were situated in various locations throughout the hospital. Screening outcomes for *bla*$_{VIM}$-bearing CPE are indicated by dots, with red representing positive results and black indicating negative results. Dashed arrows depict possible transmission events within a ward, while solid arrows represent possible transmission within a room. Two-sided solid arrows indicate probable transmission within a ward.

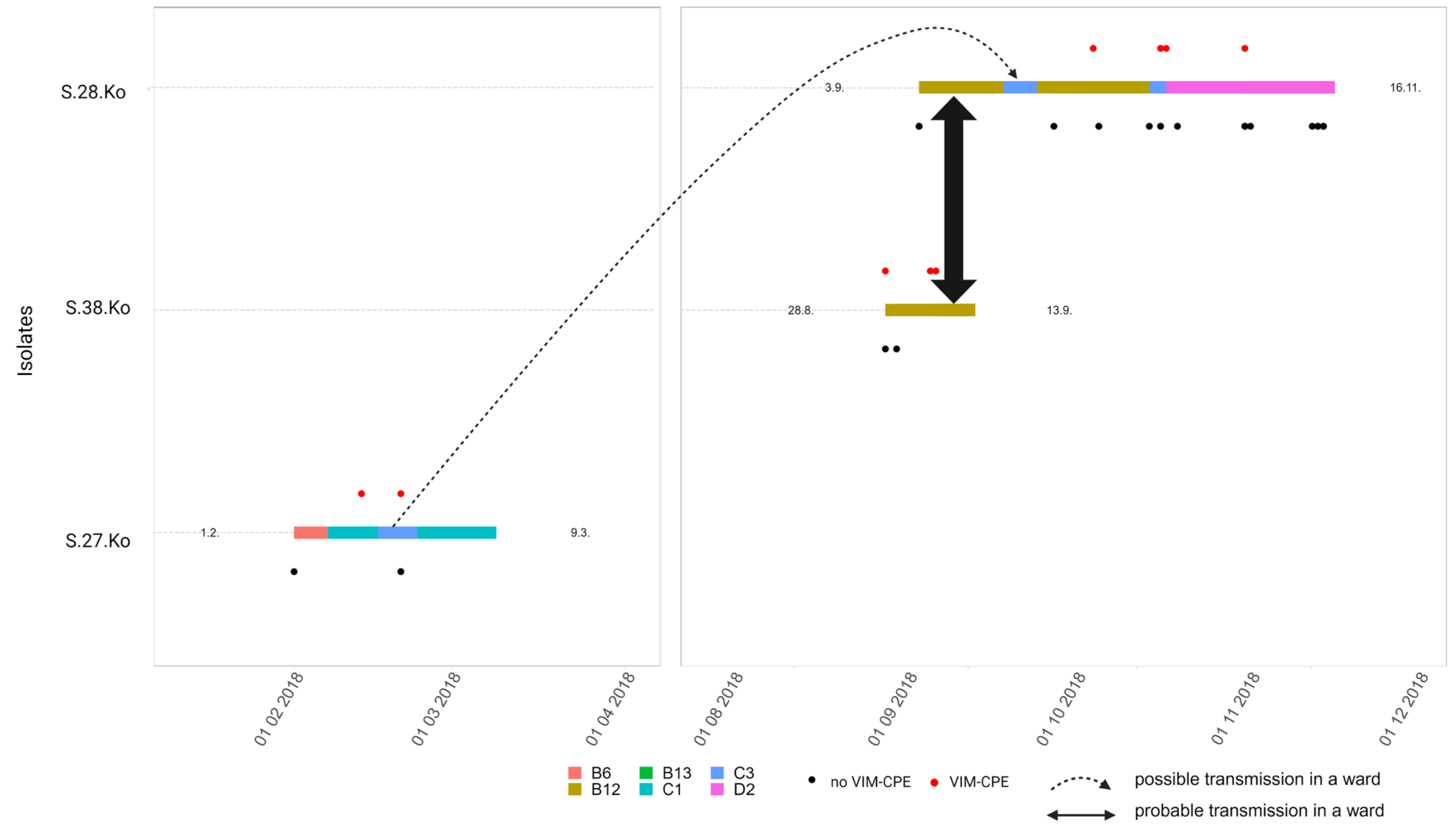

Supplementary Figure S3: Patient Movement Data and Transmission Events in Cluster 2

The figure illustrates patient movement data over time for each inpatient episode in cluster 2 (*K. oxytoca*), identified by sequencing numbers. Different colored bars represent the wards patients were admitted to, with the length of each bar indicating the length of stay. Dates adjacent to the bars denote admission and discharge. Wards were grouped into categories A, B, and C based on their proximity within the building, and then assigned random numbers. Wards labeled with a prefix of "D" were situated in various locations throughout the hospital. Screening outcomes for *bla*$_{VIM}$ -bearing CPE are indicated by dots, with red representing positive results and black indicating negative results. Dashed arrows depict possible transmission events within a ward. Two-sided solid arrows indicate probable transmission within a ward.

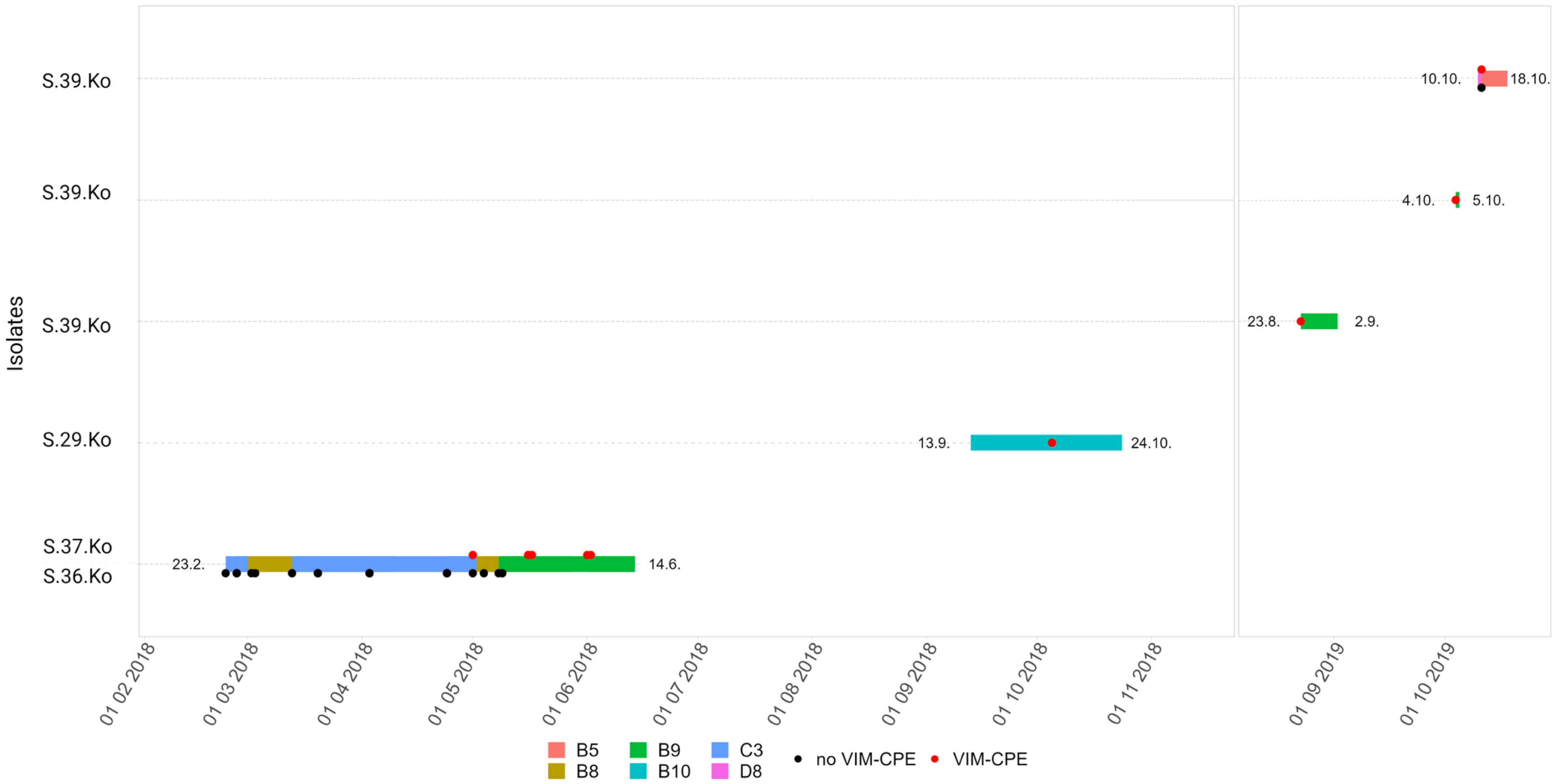

Supplementary Figure S4: Patient Movement Data in Cluster 3

The figure illustrates patient movement data over time for each inpatient episode in cluster 3 (*K. oxytoca*), identified by sequencing numbers. Different colored bars represent the wards patients were admitted to, with the length of each bar indicating the length of stay. Dates adjacent to the bars denote admission and discharge. Wards were grouped into categories A, B, and C based on their proximity within the building, and then assigned random numbers. Wards labeled with a prefix of "D" were situated in various locations throughout the hospital. Screening outcomes for *bla*$_{VIM}$ -bearing CPE are indicated by dots, with red representing positive results and black indicating negative results.

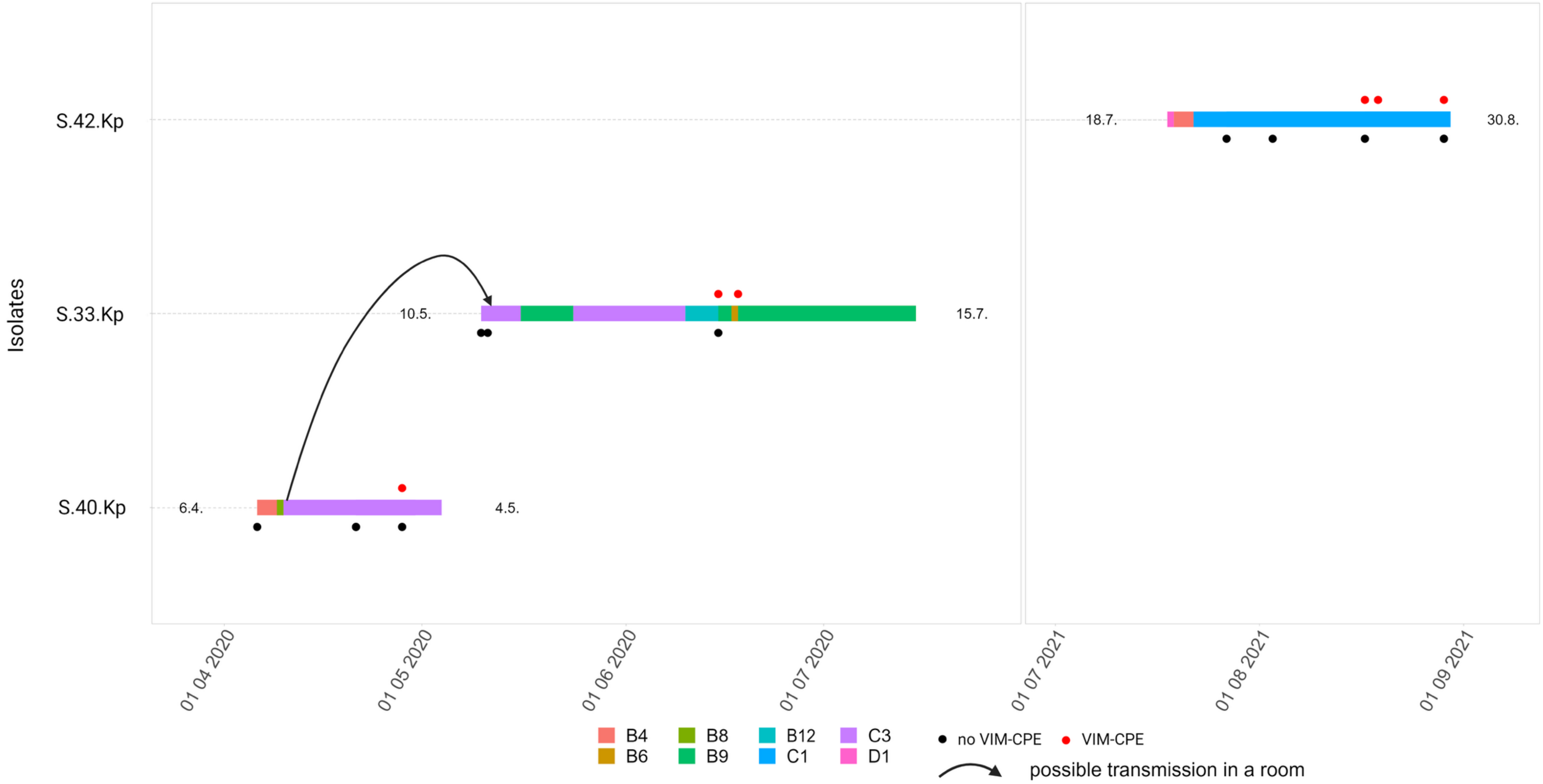

Supplementary Figure S5: Patient Movement Data and Transmission Events in Cluster 4

The figure illustrates patient movement data over time for each inpatient episode in cluster 4 (*K. pneumoniae*), identified by sequencing numbers. Different colored bars represent the wards patients were admitted to, with the length of each bar indicating the length of stay. Dates adjacent to the bars denote admission and discharge. Wards were grouped into categories A, B, and C based on their proximity within the building, and then assigned random numbers. Wards labeled with a prefix of "D" were situated in various locations throughout the hospital. Screening outcomes for *bla*$_{\mathrm{VIM}}$ -bearing CPE are indicated by dots, with red representing positive results and black indicating negative results. The arrow represents a possible transmission within a room.

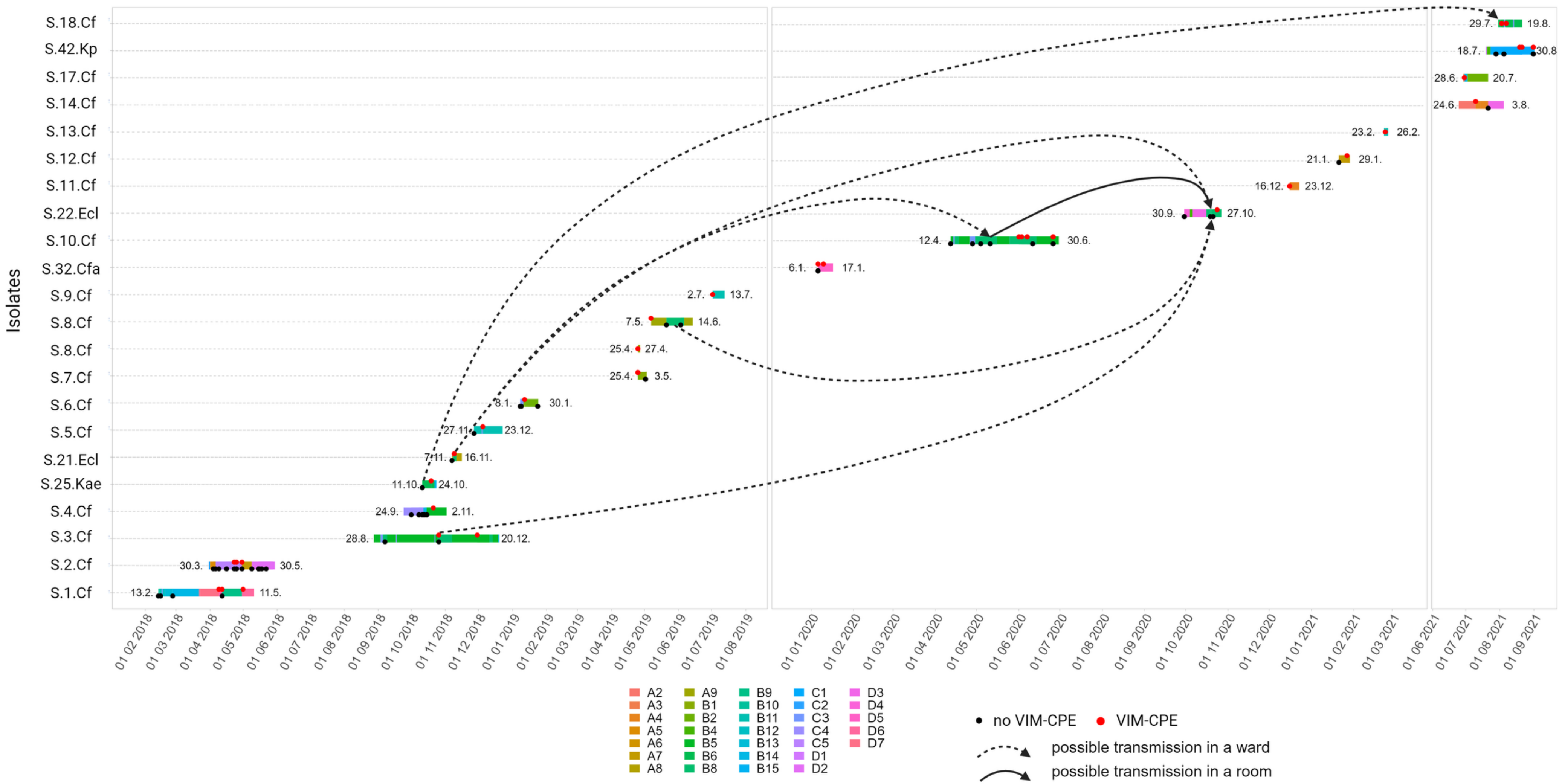

Supplementary Figure S6: Patient Movement Data and Transmission Events for Plasmid A

The figure illustrates patient movement data over time for each inpatient episode with plasmid A, identified by sequencing numbers. Different colored bars represent the wards patients were admitted to, with the length of each bar indicating the length of stay. Dates adjacent to the bars denote admission and discharge. Wards were grouped into categories A, B, and C based on their proximity within the building, and then assigned random numbers. Wards labeled with a prefix of "D" were situated in various locations throughout the hospital. Screening outcomes for *bla*$_{VIM}$ -bearing CPE are indicated by dots, with red representing positive results and black indicating negative results. Dashed arrows depict possible transmission events within a ward, while solid arrows represent possible transmission within a room.

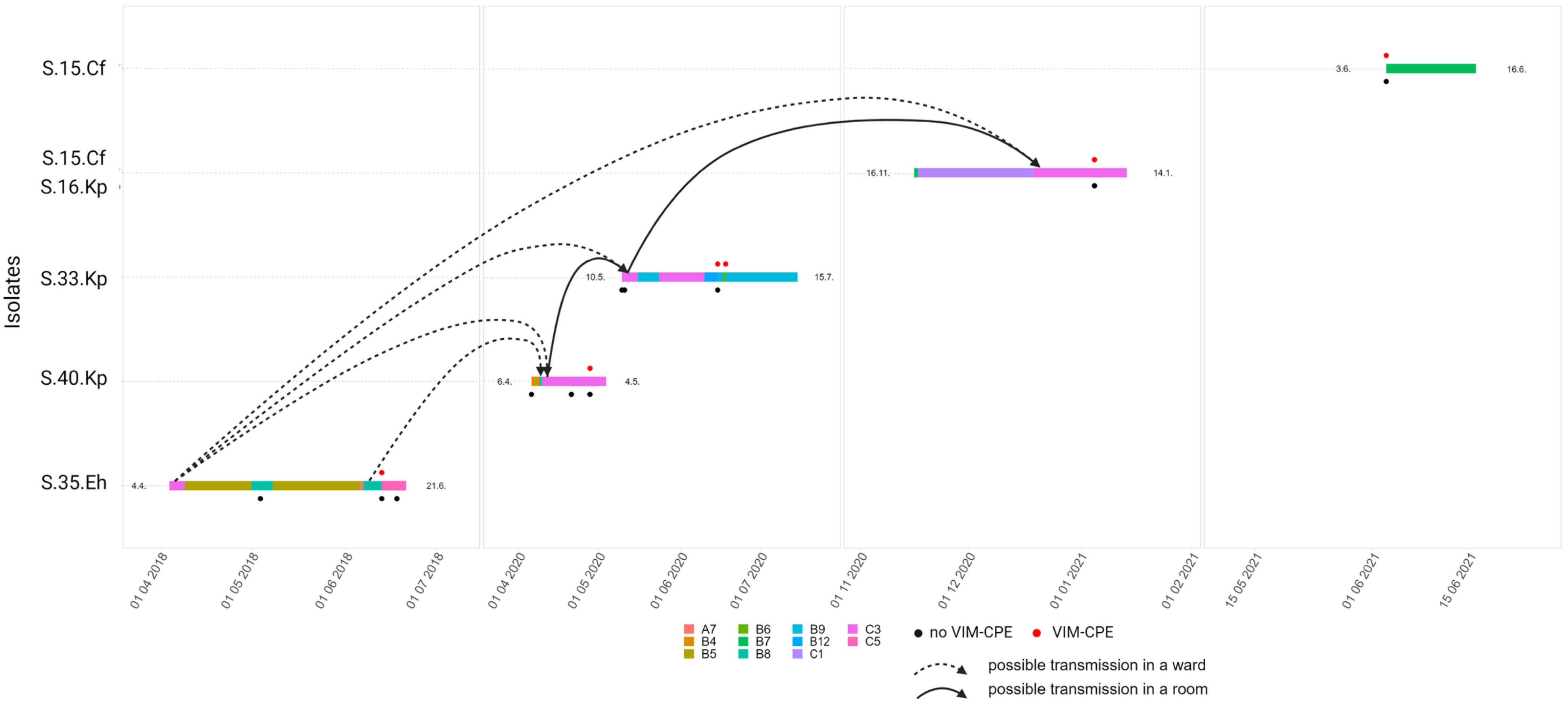

Supplementary Figure S7: Patient Movement Data and Transmission Events for Plasmid B

The figure illustrates patient movement data over time for each inpatient episode with plasmid B, identified by sequencing numbers. Different colored bars represent the wards patients were admitted to, with the length of each bar indicating the length of stay. Dates adjacent to the bars denote admission and discharge. Wards were grouped into categories A, B, and C based on their proximity within the building, and then assigned random numbers. Screening outcomes for bla_{VIM} -bearing CPE are indicated by dots, with red representing positive results and black indicating negative results. Dashed arrows depict possible transmission events within a ward, while solid arrows represent possible transmission within a room.

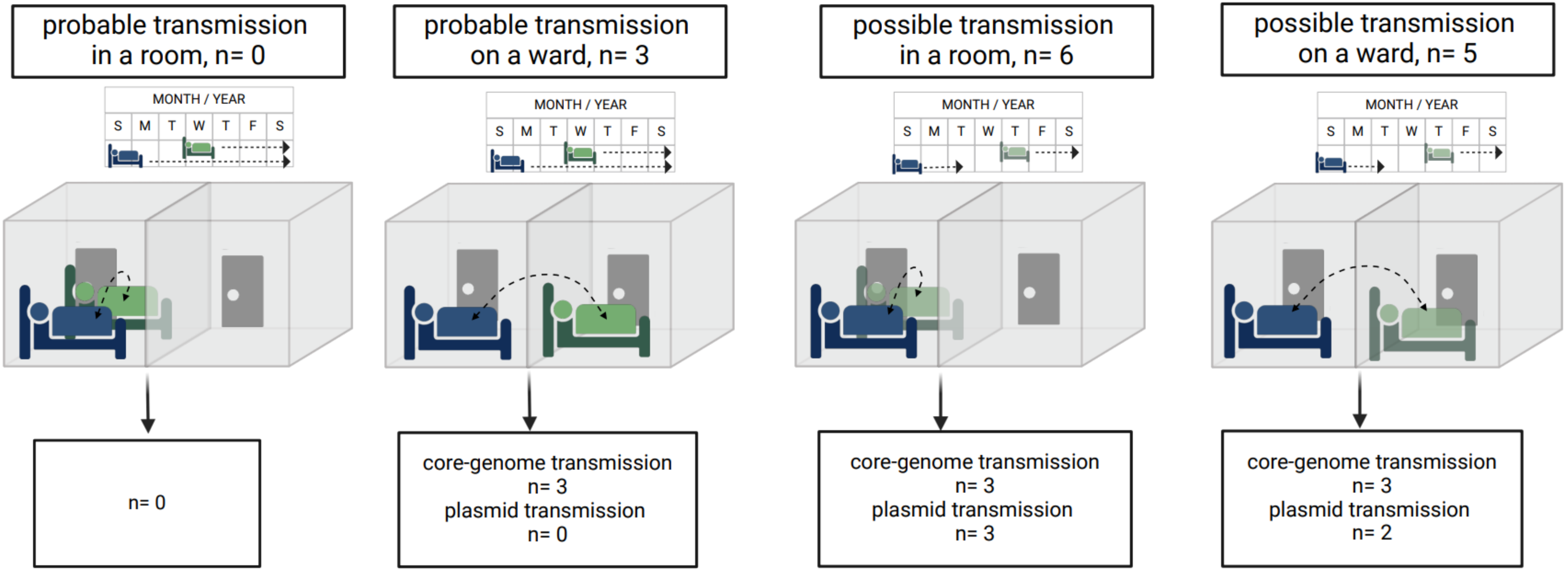

Supplementary Figure S8: Characterisation of putative transmission events for hospital-acquired isolates with genomic connections

When VIM-CPE isolates from patients had a core-genome or plasmid level connection, the patients' movements were analysed. Putative transmissions were categorised into four groups from most to least likely: 'probable transmission in a room' (transmission between patients concurrently sharing the same room), 'probable transmission on a ward' (transmission between patients concurrently admitted to the same ward), 'possible transmission in a room' (transmission between patients occupying the same room at distinct times), and 'possible transmission in a ward' (transmission between patients admitted to/in the same ward at non-overlapping times).
